# Keratoglobus: a close entity to megalophthalmos

**DOI:** 10.1186/s40064-016-2307-1

**Published:** 2016-05-17

**Authors:** Noopur Gupta, Anita Ganger

**Affiliations:** Cornea and Refractive Surgery Services, Dr. Rajendra Prasad Centre for Ophthalmic Sciences, All India Institute of Medical Sciences, New Delhi, 110029 India

## Abstract

**Background:**

Keratoglobus closely resembles buphthalmos and anterior megalophthalmos.

**Findings:**

A 45-year-old man presented with gradually progressive, painless, diminution of vision in both eyes since childhood. On examination, visual acuity of right (RE) and left eye (LE) was 20/60 and 2/20 respectively. Clinical pictures of the patient are shown in panel A, B, C, D. Keratometry values were 46.47/47.94 D at 42/132° in RE and 46.90/47.23 D at 174/84° in LE, signifying steep, ectatic cornea. Axial lengths, anterior chamber depth and corneal thickness in RE/LE was 23.53/27.12 mm, 5.18/4.48 mm and 413/420 μm respectively. Iridodonesis was noted in left eye. Retinal evaluation of LE revealed retinal detachment (RD) with posterior staphyloma due to high myopia, hereas RE was within normal limits. Intraocular pressure was normal in both eyes. Final diagnosis was keratoglobus with LE myopic RD. The patient improved to 20/30 in right eye with no improvement in LE with scleral contact lens.

**Conclusion:**

Keratoglobus, Megalophthalmos and Buphthalmos are exceedingly close entities and it is very essential to make correct diagnosis, as management options differ significantly for all three diseases.

A 45-year-old male patient presented with gradual, progressive and painless diminution of vision since childhood. On ocular examination, visual acuity of right eye (RE) and left eye (LE) was 20/60 and 2/20 respectively. Slit lamp evaluation revealed bilateral, diffuse corneal thinning in BE with outward, globular protrusion of the cornea (shown in panel A, B, C, D). The intraocular pressure (IOP) recorded with applanation tonometer was in the normal range. Keratometric values, depicting the corneal curvature, were 46.47/47.94 dioptre (D) at 42/132° and 46.90/47.23 D at 174/84° in RE and LE respectively, indicating steep and ectatic cornea bilaterally. Corneal topography assessed by Pentacam Scheimpflug Imaging system revealed, sim K values of 46.70/47.32 (steep axis at 60.9°) in RE and 45.9/47.7 (steep axis at 172.9°) in LE along with a posterior elevation of +12/+25 in RE/LE respectively. The recorded values of white-to-white diameter, axial length, corneal thickness and anterior chamber depth in RE/LE were 14.59/14.15 mm, 23.53/27.12 mm, 413/420 µm and 5.18/4.48 mm respectively. Retinal evaluation of LE revealed retinal detachment with posterior staphyloma, whereas RE was unremarkable. A diagnosis of bilateral keratoglobus with myopic retinal detachment in the LE was made. The risk of corneal perforation, even on minimal trauma was explained to the patient. On refraction no improvement in visual acuity was noted. Furthermore, the patient improved to 20/30 in the RE with a scleral contact lens while no improvement was noted in LE. The patient wilfully gave his consent to publish his medical details and images in a medical journal (Fig. 1).

**Fig. 1:**
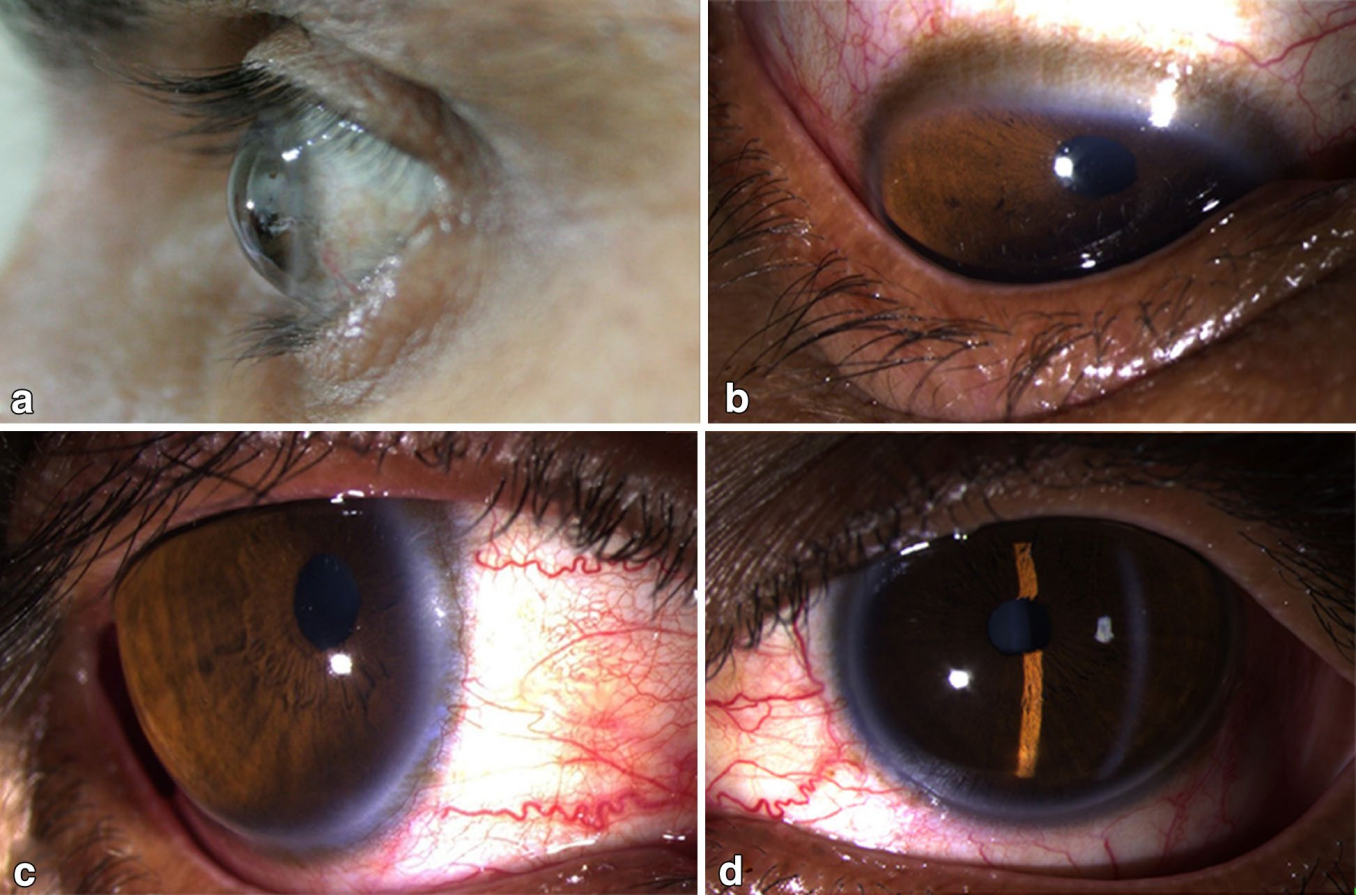
**a** Clinical picture of the patient showing diffuse thinning of cornea with outward globular protrusion. **b** Indentation of lower lids is shown on downward gaze, due to the protruded cornea. **c**, **d** Clinical pictures of the patient, showing bilateral clear cornea with deep anterior chamber with and stretched-out limbus

## Comment

Keratoglobus is a bilateral, non-inflammatory, ectatic disorder of the cornea that is characterized by globular protrusion of the cornea (Smolek and Klyce 2000). Interestingly, this disease closely resembles buphthalmos and anterior megalophthalmos, where abnormal, large eyes with enlarged cornea as well as increased axial lengths are seen in the presence and absence of glaucoma respectively (Table 1) (Dua et al. 1999). Although our patient presented with features, indicative of megalophthalmos i.e. presence of enlarged corneas, increased axial lengths, iridodonesis and absence of increased IOP (Tsai et al. 2005), but in view of steep corneal curvatures, abnormal and thin cornea, increased white to white diameter (Lockington and Ramaesh 2015), normal intraocular pressure, absence of both miosis and ciliary ring enlargement, a diagnosis of buphthalmos or anterior megalophthalmos was excluded.

**Table 1:** Enumeration of differentiating points between keratoglobus, anterior megalophthalmos and buphthalmos

	Keratoglobus	Anterior megalophthalmos	Buphthalmos/infantile glaucoma
Inheritance	No definite pattern	X-linked recessive	Sporadic
Age of presentation	Puberty	Congenital	First year of life
Natural History	Progressive	Non-progressive	Progressive
Symptoms	Frequent change of glasses	Variable and nonspecific	Watering, photophobia
Intra ocular pressure (IOP)	Normal	Normal	Elevated
Corneal diameter	>13mm; symmetric with increased WTW diameter	>13mm; symmetric	Variable, depends upon severity of glaucoma
Axial length	Increased axial length	Normal	Increased axial length
Iris	Iridodonesis ±	Iridodonesis; iris stromal hypoplasia	Normal with high insertion
Optic disc	Usually normal	Normal with increased propensity for glaucoma	Cup:disc ratio increased
Treatment	Refractive error correction and keratoplasty in advanced cases	Refractive errors correction	Control of IOP (medically/surgically)

## Conclusion

Keratoglobus, Megalophthalmos and Buphthalmos are exceedingly close entities and it is very essential to make a correct diagnosis, as management options differ significantly for all three diseases (Table 1).
